# Effects of corrosion on orthodontic mini-implants related to removal torque fracture resistance

**DOI:** 10.4317/jced.62447

**Published:** 2025-03-01

**Authors:** Júlia Dal Paz, Felipe Gomes Dallepiane, Alef da Silva, Lílian Vanessa Rossa Beltrami, William Haupt, Micheline Sandini Trentin

**Affiliations:** 1 School of Dentistry, University of Passo Fundo, Passo Fundo, RS, Brazil; 2 School of Dentistry, Federal University of Santa Catarina, Florianópolis, SC, Brazil; 3 School of Engineering, University of Caxias do Sul, Caxias do Sul, RS, Brazil; 4 School of Engineering, University of Passo Fundo, Passo Fundo, RS, Brazil

## Abstract

**Background:**

This study evaluated the effect of metallic corrosion on the torsional fracture resistance of mini-implants of different alloys in two solutions: artificial saliva and artificial saliva+fluoride.

**Material and Methods:**

The research included 60 mini-implants: 30 of Ti6Al4V and 30 of stainless steel from the brand Morelli. The groups were divided into G1: stainless steel control, G2: Ti6Al4V control, G3: stainless steel in saliva, G4: stainless steel in saliva+fluoride, G5: Ti6Al4V in saliva, and G6: Ti6Al4V in saliva+fluoride, all with n=10. A potentiostat conducted electrochemical corrosion tests. Subsequently, one mini-implant from each group underwent SEM analysis for corrosion examination (80 and 5.000x). Then, the mini-implants were removed from the rods and subjected to a mechanical torsion fracture test (500N) using a mandrel coupled to a universal mechanical testing machine. After fracture or deformation, one mini-implant from each group underwent SEM analysis again (80 and 5.000x).

**Results:**

The statistical analysis showed no significant differences between the groups (stainless steel: 0.076 and Ti6Al4V: 0.199; p*p*>0.05). The Shapiro-Wilk test indicated that the data did not follow a normal distribution (*p*<0.05). The pitting potential analysis revealed no significant differences between G3 and G4, G5 and G6, or G4 and G6. Fracture resistance tests showed that most stainless steel mini-implants deformed rather than fractured completely (G1: 33.95N; G3: 40.60N; G4: 28.26N), requiring higher force for fracture. All Ti6Al4V mini-implants fractured at lower forces due to the material’s brittleness (G2: 26.35N; G5: 27.50N; G6: 24.01N).

**Conclusions:**

All analyzed groups experienced corrosion and pitting potentials, but none exerted sufficient influence to fracture or deform the devices under torsion.

** Key words:**Mini-implants, corrosion, artificial saliva, fluoride, fracture resistance.

## Introduction

Orthodontic treatment with mini-implants has represented a highly effective anchorage method well-tolerated by patients, offering treatment possibilities that require minimal cooperation and provide maximum esthetic outcomes. The easy placement of these implants allows insertions in various locations due to their small size (1). They may be purchased in different shapes, designs, diameters, lengths, degrees of titanium alloy purity, and surface treatments. However, despite similar dimensions, they present different characteristics that may influence fracture resistance (2).

Mini-implants are manufactured from biocompatible materials, such as titanium alloys and surgical stainless steel. Titanium exhibits good corrosion resistance properties compared to stainless steel but is more expensive. Stainless steel also presents good mechanical properties, such as stiffness, ductility, and elasticity (3). The corrosion resistance of orthodontic alloys depends on their environment, as several variables may affect them, such as the quantity and quality of saliva and the pH of foods and beverages, among others (4,5).

Titanium exposure to acids, fluoridation, and saliva may remove the protective oxide film of metals, initiating a corrosion process (6). Corrosion induced by the low pH of saliva tends to increase the roughness values of metal devices. In dentistry, roughness is relevant for bacterial adhesion and colonization. Besides causing adverse health effects, ion release from corrosion may reduce material durability (6,7).

Mini-implant fracture is also a failure mode verified in several clinical studies during insertion and removal. Although this fracture is often associated with small-diameter mini-implants, corrosion seems a determinant for the fracture process. Little grooves on a mini-implant surface from corrosion may amplify the surrounding corrosive environment, compromising mechanical properties (8).

These devices promote better outcomes in critical anchorage cases, which may occasionally require position changes. Recycling mini-implants and using them in a second intervention might reduce treatment costs, potentially improving clinical outcomes. Hence, they need some torsional fracture resistance during insertion and removal. Moreover, their small size increases the likelihood of fracture during insertion and potential deformation or fracture during post-treatment removal (1,9).

Therefore, the present study evaluated the effect of metallic corrosion on the torsional fracture resistance of orthodontic mini-implants made of Ti6Al4V alloy and stainless steel in two different solutions: artificial saliva and artificial saliva with 1500 ppm of fluoride. The tested hypothesis was that, after the corrosion process, the orthodontic mini-implants of Ti6Al4V and stainless steel subjected to artificial saliva with fluoride would exhibit lower torsional fracture resistance.

## Material and Methods

The present research was a laboratory study of corrosion and fracture testing in an *in vitro* analysis. It included 60 orthodontic anchorage mini-implants from the same manufacturer: 30 Ti6Al4V mini-implants and 30 stainless steel mini-implants, both from Morelli (Morelli Produtos Odontológicos, Sorocaba, SP, Brazil). The company’s catalog aided the search for the best similarity between the mini-implants for analysis. Ti6Al4V mini-implants presented 2.0 mm in diameter, 6 mm in length, a transmucosal profile of 1.5 mm, and were manufactured from Ti6Al4V alloys. Stainless steel mini-implants exhibited 2.0 mm in diameter, 5 mm in length, a transmucosal profile of 4 mm, and were made of stainless steel alloys.

The mini-implants were divided into six groups, each consisting of 10 samples: G1 (Stainless Steel control), G2 (Ti6Al4V control), G3 (Stainless Steel in saliva), G4 (Stainless Steel in saliva with fluoride), G5 (Ti6Al4V in saliva), and G6 (Ti6Al4V in saliva with fluoride).

-Mini-implant insertions

After analyzing one mini-implant of each material under SEM, the 60 mini-implants were placed in a composite acrylic resin rod reinforced with fiberglass (Nema G10, *Pi*edmont Plastics, NC, USA) with a similar elastic modulus (16 GPa) to the human cortical bone (10). These rods were standardized with circumferences of 10 mm and depths of 5 mm to ensure complete mini-implant insertion, exposing the thread apices for possible corrosion testing. A specific drill from the Morelli mini-implant insertion kit drilled the Nema G10 rods. An insertion key attached to a 20:1 reduction contra-angle in a surgical motor (both from Neodent, Curitiba, PR, Brazil), under irrigation, aided mini-implant placements in the Nema G10. The rotation per minute and torque followed the manufacturer’s recommendation (50 rpm and 25N). This insertion procedure was interrupted when the mini-implant was locked in the Nema G10, preventing the motor from rotating. A manual torque key allowed complete mini-implant insertion into the Nema G10 when necessary.

-Corrosion analysis

The NatuPharma compounding pharmacy (Passo Fundo, RS, Brazil) prepared artificial Fusayama saliva, the electrolyte solution of this *in vitro* study. This solution was initially fluoride-free and subsequently included a fluoride concentration of 1500 ppm, replacing the saliva in each sample for testing. The electrochemical behavior of metallic materials in this solution was similar to that in human saliva (11).

The Fusayama artificial saliva solution is composed of sodium chloride (NaCl) at 0.4 g/L, potassium chloride (KCl) at 0.4 g/L, calcium chloride dihydrate (CaCl2•2H2O) at 0.795 g/L, sodium sulfide nonahydrate (Na2S•9H2O) at 0.005 g/L, sodium phosphate monohydrate (NaH2PO4•2H2O) at 0.69 g/L, and urea at 1 g/L. The applied fluoride concentration considers the same concentration in the oral cavity, with toothpaste containing fluoride concentrations of up to 1500 ppm.

The corrosion test had copper wires threaded at mini-implant apices. The other end of these wires was threaded into the copper clamp attached to the potentiostat, leaving only the transmucosal portion and the head of mini-implants exposed to electrolytic solutions. Beeswax isolated the copper devices from the electrolytic substances to prevent interference with the corrosion test. Moreover, polarization procedures occurred inside a Faraday cage to isolate the system from external electromagnetic waves, preventing interferences and ensuring a more reliable outcome.

The electrochemical tests used the IVIUMSTAT potentiostat coupled to Ivium A11701 software for electrochemical control and data analysis. This equipment generated an open-circuit potential (OCP) defined as the potential of a conductor immersed in an ion-conducting electrolyte measured against a reference electrode. The potentiostat allowed the imposition of the desired potential on the working electrode relative to the reference electrode, measuring and recording the polarization current according to potential using a recorder (12).

After immersing the sample in the electrolytes (saliva and saliva+fluoride), the OCP evolved, adding a one-hour waiting time for each sample until the OCP stabilized. Corrosion requires a lower equilibrium potential of the anodic metal dissolution reaction than that of the hydrogen reduction reaction (cathodic) (12). The test applied cathodic polarization by increasing voltage until reaching pitting corrosion. *Pi*tting corrosion consists of a highly localized metal attack only in a specific medium at electrode potentials equal to or greater than a given potential, known as the pitting potential (12).

At the end of the polarization test, all samples repeated cleaning procedures with running water and mini-implant storage to prepare for surface analysis. The computer provided numbers such as current density and applied potential. Ivium A11701 software measured the area of the mini-implant head in square centimeters (cm2), determining the corrosion area. The current/area calculation measured current density, and these data generated Excel graphs using a logarithmic scale for descriptive analysis.

The Shapiro-Wilk normality test conducted normality testing, and the statistical analysis used the Kruskal-Wallis test and Student-Newman-Keuls comparison to evaluate corrosion and pitting potentials.

After the corrosion tests, one sample from each group was subjected to SEM analysis again before the fracture test. The analysis equipment was from the TESCAN brand, model LM3 Vega (Curitiba, PR, Brazil). The surface of sample heads was analyzed and photographed at 80x, 800x, and 5000x magnification to verify cracks or corrosion of materials (13). The obtained images allowed sample comparisons, selecting one per group to explain corrosion.

-Removal torque fracture test

Torque fracture tests also examined samples from each control group (n=10 for stainless steel mini-implants and n=10 for Ti6Al4V mini-implants).

The manual key from the Morelli insertion and removal kit aided in mini-implant removal from the Nema G10 rods.

As no mini-implant fractured during the removal test with the manual key, all of them endured a mechanical torsion fracture test using a mandrel attached to a universal mechanical testing machine (Shimadzu, Barueri, SP, Brazil) with a 500N load cell. As for torsion fracture, the mandrels clamped both ends of the mini-implant. One mandrel was fixed at the attachment of the mini-implant tip, and the other rotated by pulling a polymer wire attached to the shaft and the load cell holding the mini-implant head. Considering one fixed end and the other rotating, a torque force was generated on the mini-implant, and the Trapezium X program (Shimadzu, Barueri, SP, Brazil) recorded it as the maximum force upon fracture. The software calculated the fracture torque by multiplying the maximum force by the radius of the axis where the polymer wire was wound, according to the equation: Torque (T) = Force (F) x 4. All numerical results were presented as mean and standard deviation.

-Visual surface analysis under scanning electron microscope (SEM)

After mini-implant fracture or deformation, one implant from each group returned to SEM analysis. The mini-implants were photographed at 80x and 5000x magnification to verify potential corrosion signs in the region of fracture or deformation.

## Results

-Corrosion test

The corrosion test presented values in current density (A/cm²) and potential (V). It is worth noting that this study only analyzed the potential (V).

The Shapiro-Wilk normality test demonstrated that data did not follow a normal distribution ( Table 1) (*p*<0.05). The statistical analysis evaluating pitting potential and comparing the test groups ( Table 2) did not show significant differences between groups G3 and G4, G5 and G6, and G4 and G6. There was a difference between G3 (stainless steel in saliva) and the others, as G3 required a lower potential to form corrosion pits. The statistical analysis for evaluating corrosion potential and comparing the groups ( Table 2) did not present significant differences between G3 and G4 nor between G5 and G6. There was a significant difference between G4 (stainless steel in saliva+fluoride), which formed the corrosion potential the fastest, and G6 (Ti6Al4V in saliva+fluoride), which took the longest to form the corrosion potential. G3 (stainless steel in saliva) and G6 (Ti6Al4V in saliva+fluoride) also differed. The materials and solutions to which the groups were exposed explain this difference.

-Torsion fracture test results

Torsion fracture resistance tests showed that most stainless steel mini-implants (G1, G3, and G4) only deformed instead of completely fracturing. Furthermore, mean, median, and standard deviation values demonstrated the need for higher force application for such effects. Conversely, all Ti6Al4V mini-implants (G2, G5, and G6) fractured under lower forces ( Table 3), likely because of their more brittle material. Considering that data did not follow a normal distribution, as indicated by the Shapiro-Wilk test, the Kruskal-Wallis statistical analysis was performed, not showing significant differences between groups, including when compared to the control groups of each material.

**Figure 1:**
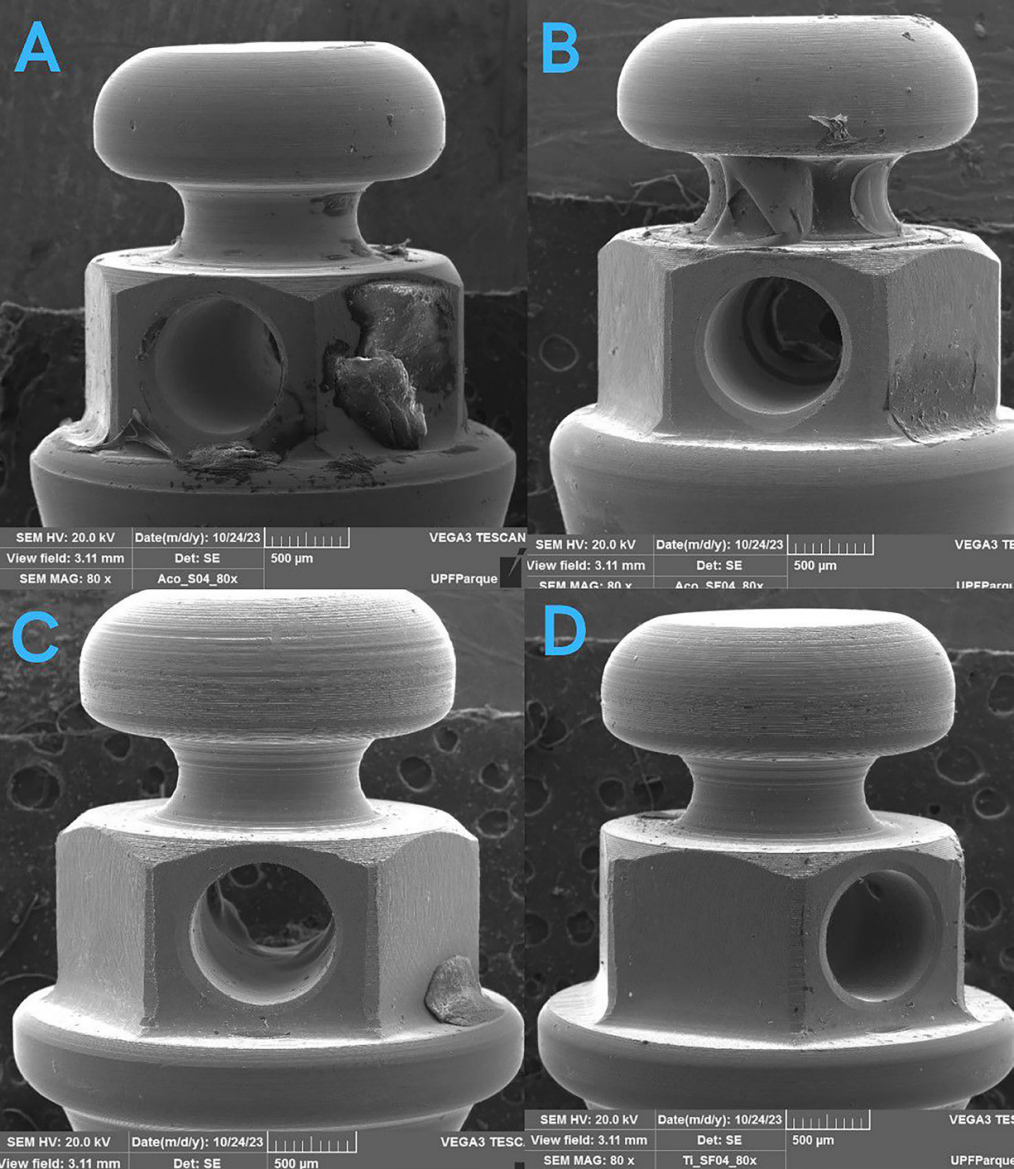
Mini-implants of A (Stainless steel in saliva), B (Stainless steel in saliva + fluoride), C (Ti6Al4V in saliva), D (Ti6Al4V in saliva + fluoride) before the fracture test.

**Figure 2:**
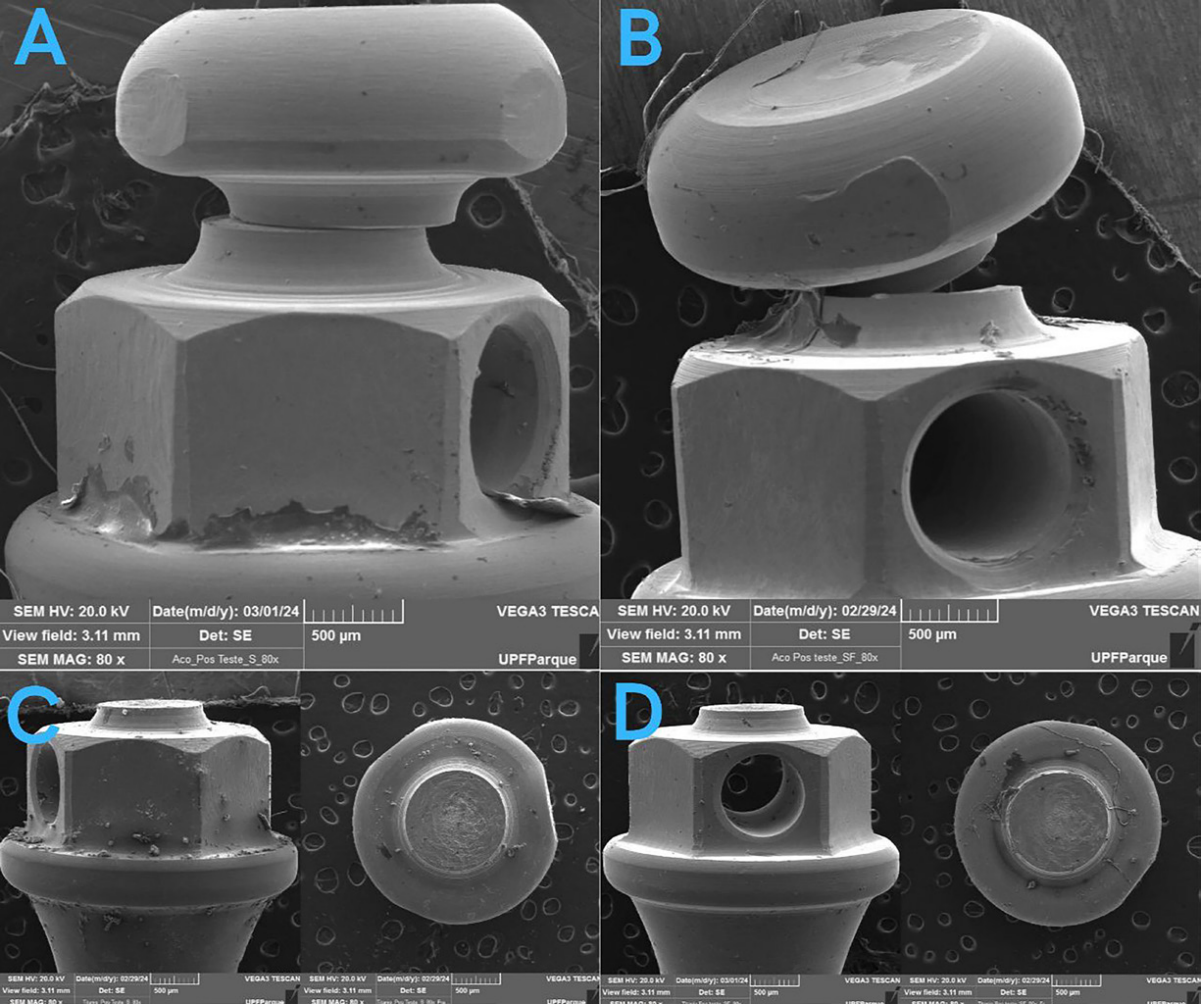
Mini-implants of A (Stainless steel in saliva), B (Stainless steel in saliva + fluoride), C (Ti6Al4V in saliva), D (Ti6Al4V in saliva + fluoride) after the fracture test.

Photographs at 80x magnification of the mini-implants under a SEM before and after the fracture test showed no signs of corrosion in the regions of fracture or deformation. These photographs reveal that the stainless steel devices only deformed, whereas the Ti6Al4V devices completely fractured at their most fragile portion, which was the neck (Figs. 1,2).

## Discussion

The current market offers mini-implants manufactured with biocompatible elements such as titanium alloys and surgical stainless steel (3,14). The fracture of these devices during insertion or removal remains among the most commonly reported complications by orthodontists (15,16). The corrosion of orthodontic metal alloys is another complication of mini-implant manufacturing materials (4,5,8). Therefore, evaluating whether corrosion alters fracture resistance during material removal is relevant, testing in a medium simulating the oral cavity with artificial saliva and fluoridated toothpaste for oral hygiene control.

The information from the articles mentioned demonstrated a variation in the parameters of corrosion studies. The tested solutions simulated the physiological solutions of the human body (14,17–20). Our study showed that saliva and fluoride produced corrosion in the tested alloys but without significant differences between the solutions. Hence, further *in vivo* studies are required, considering that other factors in the oral cavity may influence this corrosion, such as diet, hygiene, and peri-implant toxicity.

The corrosion tests in this study demonstrated that the mini-implants manufactured with Ti6Al4V alloy and stainless steel from the brand Morelli were susceptible to corrosion. The mini-implants in G6 (Ti6Al4V in artificial saliva+fluoride) showed higher resistance potential to corrosion and pitting corrosion. That is because fluoride in contact with titanium alloys harms the properties of the titanium oxide layer and its alloys (6).

Titanium and its alloys provide higher corrosion resistance in saline and acidic environments. Titanium may be as corrosive as other primary metals when the stable oxide layer is broken or removed and becomes unable to reform on parts of the surface (13). Stainless steel contains components such as iron, chromium, and nickel, which release corrosion products that form highly acidic chloride solutions, promoting high corrosion rates in surrounding tissues (21).

Mini-implant diameters significantly impact fracture torque values. Thus, mini-implants with larger diameters may be beneficial, added by higher primary stability (1,22–24). However, this study tested Ti6Al4V and stainless steel mini-implants with the smallest available size (diameter and height) precisely to test them under challenging conditions.

Stainless steel is less expensive than Ti6Al4V and presents good mechanical properties, such as stiffness, ductility, and elasticity, meeting the minimum criteria for an excellent mini-implant (3,25). These stainless steel features agree with the present study, showing that most mini-implants of this material only deformed instead of completely fracturing. They also required higher force in Newtons to achieve such effects.

The literature describes that stainless steel mini-implants may trigger more bone damage or even screw loss because they support higher torque, not achieving favorable osseointegration due to more intense bone compression and causing microfractures (2). That aligns with the present study because, despite suggestive corrosiveness in the samples, there was no confirmation that it causes device failure and impairs clinical function alone, as we also tested the control group of each material (Ti6Al4V and stainless steel) in the fracture test. The mini-implants from control groups, which had not undergone the corrosion test, fractured similarly, requiring the same torsional force as the other groups, whose mini-implants had been subjected to corrosion in the electrolytic solutions.

The hypothesis that Ti6Al4V and stainless steel orthodontic mini-implants subjected to artificial saliva with fluoride would present lower torsional fracture resistance after the corrosion process was rejected. The relevance of this study is the clinical correlation between the corrosion of different metal alloys for manufacturing orthodontic mini-implants and the possibility of their fracture when applied with a force equivalent to their removal torque. The study limitations were *in vitro* testing and the absence of other variables in the oral cavities of different individuals.

## Conclusions

All groups studied in the corrosion analysis experienced corrosion and pitting potentials, but none exerted sufficient influence to fracture or deform the devices under torsion testing. Torsion fracture tests showed similar resistance for both groups. Stainless steel demonstrated excellent resistance to deformation, whereas Ti6Al4V promoted deformation followed by fracture. Therefore, orthodontists should control other factors, such as selecting the ideal mini-implant design for the placement site, surgeon’s skill, and patient hygiene care to achieve better success rates.

## Figures and Tables

**Table 1:** Shapiro-Wilk normality test. The groups indicated with (*) do not exhibit data normality according to the Shapiro-Wilk test (*p*<).

Pitting Potential (V)	Group 3: Steel+ saliva	Group 4: Steel+ saliva&fluoride	Group 5: Ti6Al4V+ saliva	Group 6: Ti6Al4V+ saliva&fluoride
	x̄̄(s)	x̄̄(s)	x̄̄(s)	x̄̄(s)
	0.87 (0.26)	1.30 (0.13)	1.93 (0.20)	1.97 (0.13)
p-value	0.446	0.025*	0.394	0.526
Corrosion Potential (V)	Group 3: Steel+ saliva	Group 4: Steel+ saliva&fluoride	Group 5: Ti6Al4V+ saliva	Group 6: Ti6Al4V+ saliva&fluoride
	x̄̄(s)	x̄̄(s)	x̄̄(s)	x̄̄(s)
	0.40 (0.78)	0.33 (0.15)	0.68 (0.14)	0.73 (0.15)
p-value	0.220	0.024*	0.151	0.014*

**Table 2:** Pitting and corrosion potentials. Means followed by the same uppercase letters in the rows did not differ according to the Kruskal-Wallis test (*p*>).

Pitting Potential (V)	Group 3: Steel+ saliva	Group 4: Steel+ saliva&fluoride	Group 5: Ti6Al4V+ saliva	Group 6: Ti6Al4V+ saliva&fluoride	p-value
	x̄̄	x̄̄	x̄̄	x̄̄	
	0.87 ^A^	1.30 ^AB^	1.93 ^BC^	1.97 ^C^	< 0.001
Corrosion Potential (V)	Group 3: Steel+ saliva	Group 4: Steel+ saliva&fluoride	Group 5: Ti6Al4V+ saliva	Group 6: Ti6Al4V+ saliva&fluoride	p-value
	x̄̄	x̄̄	x̄̄	x̄̄	
	0.40 ^A^	0.33 ^A^	0.68 ^B^	0.73 ^B^	< 0.001

**Table 3:** Mean, median, and standard deviation values from fracture tests in each group (n=10). Fracture force was measured in Newtons (N).

	Group 1: SSteel control	Group 2: Ti6Al4V control	Group 3: SSteel in saliva	Group 4: SSteel in saliva+fluoride	Group 5: Ti6Al4V in saliva	Group 6: Ti6Al4V in saliva+fluoride
	n=10	n=10	n=10	n=10	n=10	n=10
Mean	33.95 N	26.35 N	40.60 N	28.26 N	27.50 N	24.01 N
Median	35.18 N	25.79 N	35.00 N	28.02 N	27.71 N	24.17 N
Standard-deviation	6.957	7.780	14.310	6.322	5.176	4.148

## Data Availability

The datasets used and/or analyzed during the current study are available from the corresponding author.
